# Point-of-Care Tests for Hepatitis B Are Associated with A Higher Linkage to Care and Lower Cost Compared to Venepuncture Sampling During Outreach Screenings in an Asian Migrant Population

**DOI:** 10.5334/aogh.2848

**Published:** 2020-07-16

**Authors:** Erwin Ho, Peter Michielsen, Pierre Van Damme, Margareta Ieven, Irene Veldhuijzen, Thomas Vanwolleghem

**Affiliations:** 1 Department of Gastroenterology and Hepatology, Antwerp University Hospital, Antwerp, BE; 2 Laboratory of Experimental Medicine and Paediatrics, University of Antwerp, Antwerp, BE; 3 VAXINFECTIO, Vaccine and Infectious Diseases Institute, University of Antwerp, Antwerp, BE; 4 Division of Infectious Disease Control, Municipal Public Health Service Rotterdam-Rijnmond, Rotterdam, NL; 5 Department of Gastroenterology and Hepatology, Erasmus University Medical Centre, Rotterdam, NL

## Abstract

**Background:**

This study compares venepuncture versus point-of-care (POC) HBsAg tests on screening cost and linkage to care in prospective outreach screenings in an Asian population in three major cities in Belgium between 10/2014 and 5/2018.

**Methods:**

Two community outreach screening programs were organised between 10/2014 and 5/2018. The first screening program used venepuncture and serologic testing for HBsAg. In the second program, HBsAg was tested in finger stick blood POC tests. Positive results were confirmed during outpatient visits with serologic testing. Linkage to care was defined as having received specialist care follow-up with at least one abdominal ultrasound within three months of screening.

**Results:**

For 575 participating individuals, 571 valid results were obtained, 456 with venepuncture, and 115 using POC testing. Overall HBsAg seroprevalence was 6.8%. Linkage to care was higher when using POC testing compared to venepuncture (86% or n = 6/7 versus 34% or n = 11/32; p = 0.020). The POC screening program was economically more attractive with a total cost of € 1,461.8 or € 12.7 per person screened compared to € 24,819 or € 54.0 per person screened when using venepuncture testing. Results and an appointment for specialist care follow-up were given onsite with POC testing, while with venepuncture testing; results were sent within 20–45 days.

**Conclusion:**

In an Asian migrant population in Belgium with an HBsAg seroprevalence of 6.8%, HBV screening based on POC tests resulted in lower costs per person screened (76.5% lower), and higher linkage to care (2.5 times).

## Introduction

Viral hepatitis remains a major, worldwide health issue. Recent reports from the World Health Organization [1,2] estimate that 1.34 million deaths occur yearly by viral hepatitis B and C infections and its sequelae. Testing guidelines and a call to eradicate chronic viral hepatitis by 2030 have been recently released by the WHO, underpinned by advances in diagnostics and the availability of effective treatment [3,4]. Screening for HBV is cost-effective in migrant populations [10], but the linkage to care is a common challenge in these initiatives. Of all migrant populations in the European Union as a whole, individuals from Chinese descent make up the highest number of estimated HBV infected patients, with HBV seroprevalences of 6.2%–8.7% [5,7,8].

Screening of this hard-to-diagnose population has often been performed by community outreach methods, wherein medical staff performs screening on-site, cooperating with Chinese communities [6,9]. The Centres for Disease Control’s Hepatitis Testing and Linkage to Care (HepTLC) project showed that less than half of individuals who tested positive for HBsAg attended a first medical appointment [11]. Multiple barriers to care exist in several infectious diseases, including in hepatitis B infection care [12]. In sexually transmitted infections and HIV care, point-of-care tests (POCT) have been shown to overcome most of these barriers to care, but their impact on linkage to care in viral hepatitis B remains undetermined [13]. Recently, rapid POCT has become available for HBsAg. The performance characteristics of certain tests are excellent with sensitivities of 99.9% and specificities of 99.8% [14,15].

## Methods

This study aims to compare screening costs and linkage to care for viral hepatitis B infection in a migrant population with high expected seroprevalences using POCT or standard venepunctures during outreach screening activities.

Between 10/2014 and 5/2018, community screening programs were organised with the cooperation of Chinese community leaders, Chinese organisations and the Antwerp City Council. This allowed us to screen the same Asian population through multiple angles: during community events in various locations (churches, temples, and a local public library in Antwerp and adjacent major Belgian cities), in Asian massage parlours in Antwerp, and through opportunistic testing during mandatory integration classes.

### Patient and Public Involvement

Community leaders, volunteers, and screened persons of the Chinese community all participated before, during, and after the various screening events. Preparatory meetings were organized to disseminate the rationale of the study and make arrangements for the logistics of the screening events. A pre-screening questionnaire, communication through written media, social media, and word-to-mouth were used. During screening events, community members, translators, and Antwerp City Council social workers assisted in logistics and translation services. After screening events, community members also assisted in translation during telephone calls for results and to set up outpatient appointments.

Venepuncture testing for HBsAg was subsequently performed in the Antwerp University Hospital laboratory (Elecsys HBsAg II, Roche Diagnostics GmbH, Mannheim, Germany). POC testing for HBsAg was done with Vikia HBsAg tests (Biomérieux SA, Marcy-l’Etoile, France), according to the package insert. Testing was performed pseudonymised (no personal details were provided but results were given to the screened person) to maximize screening uptake in this difficult-to-reach population. Serologic results were given on-site after an incubation time of 15 minutes for the Vikia test, as per the package insert. Upon receiving a positive result, patients were identified and an appointment for specialist care follow-up was immediately agreed upon. POC tested patients’ results were confirmed using the standard of care serologic testing (Elecsys HBsAg II, Roche Diagnostics GmbH, Mannheim, Germany) at their first outpatient visit.

Inclusion criteria were: being of Asian descent, first- or second-generation migrant and birth date before 1999, which is the year of starting universal HBV vaccination in Belgium. Excluded were individuals <18 years old at the screening event and third-generation migrants. Minimal clinical data were obtained after informed consent. Data obtained included: age, gender, nationality, place of birth, HBV infection status, HBV vaccination status, and previous or ongoing anti-viral treatment. Results were sent through the mail and positively screened persons were notified by phone and also invited for specialist care follow-up.

Additional reflex biochemical (ALT) and virological tests were performed on HBsAg positive samples (HBeAg and anti-HBe antibody: Liaison, DiaSorin, Saluggia, Italy; HBV DNA: in-house method).

Screening uptake was defined as participating with the screening programs when reached (in any of the described screening events) and providing a valid serological result. Linkage to care was defined as having received specialist care follow-up within three months with HBsAg, HBeAg, anti-HBe antibody, ALT, and HBV DNA test results, and at least one abdominal ultrasound. Analysis of linkage to care was finalised three months after the last screening activity by cross-checking positively screened persons and the presence of out-patient clinic patient files at the Antwerp University Hospital. Treatment and hepatocellular carcinoma (HCC) surveillance indications were based on international clinical practice guidelines [23,24]. All costs associated with the screening activities (personnel, screening tests, other test materials, event-related costs such as fees for screening locations, and communication costs) are listed in the euro. Tests performed in the follow-up were excluded, as they were not billed to the patients. Turn-around times, or the amount of time passed between venepuncture and posting of results in the venepuncture, were calculated in days.

### Statistics

Before the study, a power analysis was performed to ascertain a valid sample size for the seroprevalence of HBV. Previous studies of similar scope [5,6,7] report a prevalence of HBV infection of 8.5–8.7%. Considering estimates of the Chinese population in Belgium of approximately 13,000–23,000 individuals [16] and extrapolation of 6,239 East Asians in Antwerp city proper in 2014, a power analysis with a precision of 5% (confidence interval of 95%) yielded a required sample size of n = 119.

To determine differences in sampling, the seroprevalence of HBsAg between the two screening programs was compared using the Chi-square test. To compare screened persons’ characteristics in terms of linkage to care, students’ T-tests were used to compare continuous variables. Chi-square or, where applicable, Mid-P exact test was used to compare ordinal and categorical variables, including the association between screening programs and linkage to care. Given the absence of estimates on differences in linkage to care for both screening programs, no a priori power calculation thereof was performed.

Statistical tests were performed using SPSS version 24.0 (IBM Corp. Released 2016. IBM SPSS Statistics for Windows, Version 24.0. Armonk, NY: IBM Corp.).

## Results

### Organisation of screening

Seven events were held between 10/2014 and 12/2015 (Antwerp: n = 4; Brussels: n = 2 and Leuven n = 1). Both religious (two Christian fellowships/churches, a Buddhist temple) and non-religious focus groups (Hong Kongese and other Chinese societies) were involved. Subsequently, thirteen events were held between 12/2015 and 5/2018 in Antwerp, in 23 massage parlours, five events in classrooms of the Atlas Integration and Citizenship Education organization, and one time at the Antwerp University Hospital. Details are listed in Table 1.

**Table 1:** Screening protocols and characteristics.

Screening Protocols and Characteristics	Venepuncture	Point-of-care test

Cooperating partners	Chinese community – Key opinion leaders	City of Antwerp/Atlas Integration and Citizenship Education/Church
Number of hospital staff	4–7	1
Number screening events	7	13
Number screening sites	5	27
Number of screened persons	460	115

### Epidemiology, Demographics

**Figure 1:**
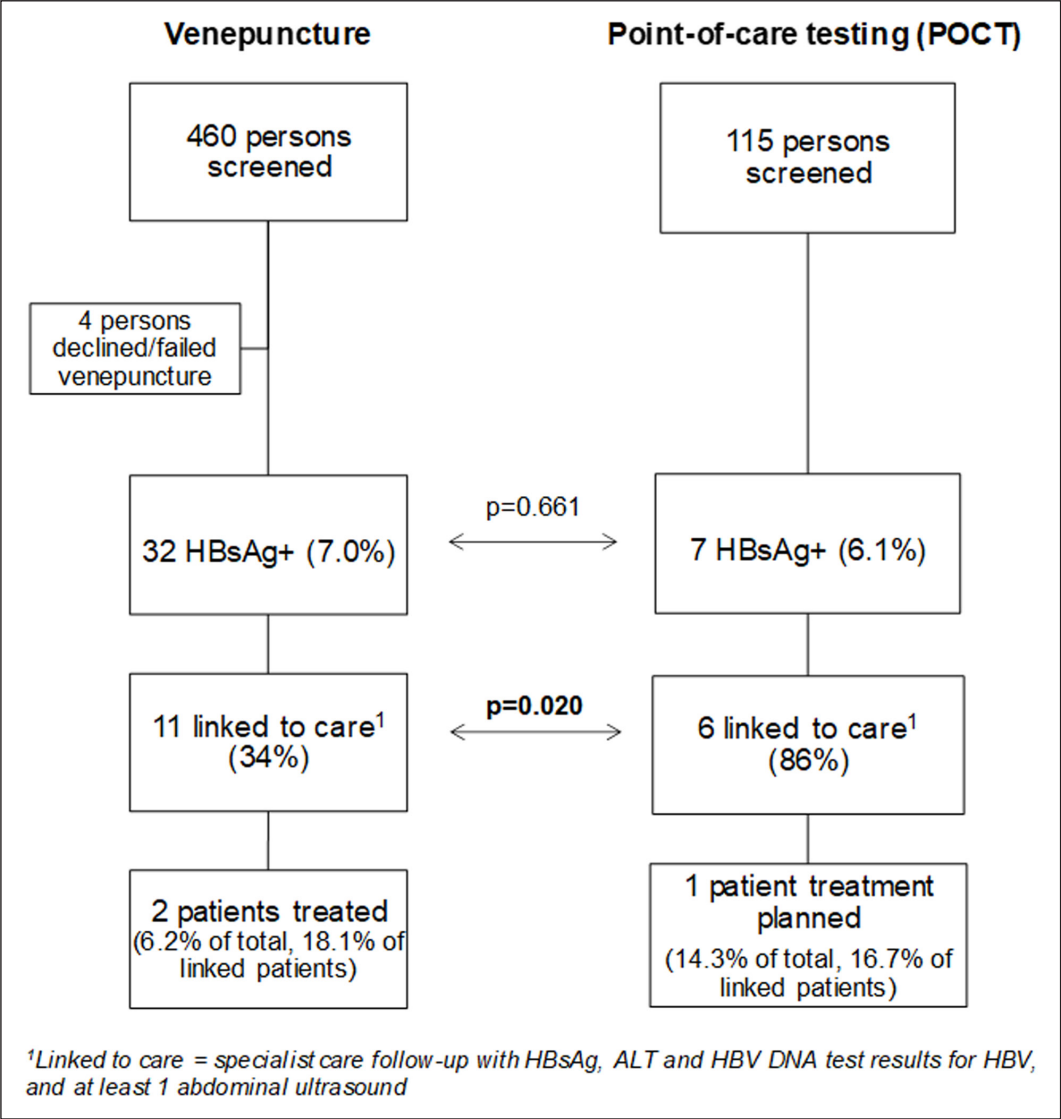
Overview of linkage to care of community screening using venepuncture and point-of-care testing (POCT).

In total, 575 persons participated, and 571 individuals were serologically screened. Using venepuncture, 456 persons had valid serologic results, and 115 persons were screened using POC testing. Overall, seroprevalence for HBsAg was 6.8% (39/571), with 32 and 7 individuals testing positive by venepuncture and POCT respectively. There was no statistically significant difference in HBsAg seroprevalence in both screening methods (7.0% vs 6.1%, p = 0.661). An overview is shown in Figure 1. One person who tested positive for HBsAg was a second-generation migrant. This person was born before 1999, before universal vaccination in Belgium and also before generalised HBsAg pregnancy testing in 1982.

### Impact of point-of-care testing: comparison of linkage to care, cost

**Figure 2:**
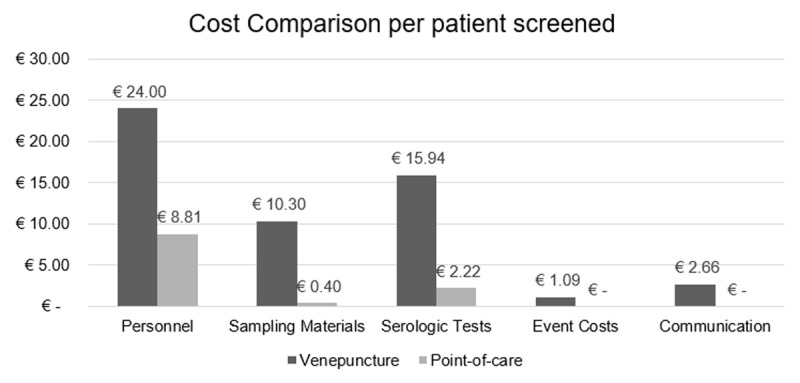
Turn-around time (time-to-result in days) for results of venepuncture screening.

The average turnaround time was 32 days (20–45 days, Figure 2) for venepuncture samples. Results were immediately available with POC testing. Eleven of the 32 HBsAg positives diagnosed using venepuncture (34%) were linked to specialist care, compared to six of the seven positive patients diagnosed with POC testing (86%) (Figure 1; p = 0.020). As we collected demographic data in the total venepuncture cohort after informed consent and in those linked to care for POCT, we were able to analyse differences in demographics between patients linked to care in both programs, as well as those not-linked to care in the venepuncture program: Age, gender, country of origin and place of residence were not associated with linkage to care, or the absence thereof in the venepuncture program (Table 2). There was no difference in the proportion of patients who met HCC surveillance criteria or treatment indications. Nor was there a difference in ALT and HBV DNA elevation between linked and non-linked patients in the venepuncture program. Ten (25.6%; 10/39) and three (7.7%; 3/39) of identified HBV patients met criteria for HCC surveillance or treatment initiation respectively [23] but were lost to follow-up (Table 2). Two patients screened with venepuncture are currently being treated with antiviral therapy according to their natural history and international treatment recommendations. For one patient screened with POC testing, antiviral treatment is being planned after a liver biopsy [21,22]. None of the patients who were linked to care tested positive for anti-Hepatitis C antibodies or anti-hepatitis Delta antibodies.

**Table 2:** Characteristics of screened persons not linked to care/linked to care.

	All (n = 39)	Not linked to care^1^ (n = 22)	Linked to care (n = 17)	p Value

**Demographics**				
Age (mean, years)^2^	46.5	46.8	45.1	0.12
Gender (female, %)	22 (56.4)	12 (54.5)	10 (58.8)	0.79
Country of origin (China, %)	38 (97.4)	22 (100)	16 (94.1)	0.25
Residence (Antwerp, %)	19 (48.7)	10 (45.5)	9 (52.9)	0.64
**Type of screening (POCT,^6^ %)**	7 (17.9)	1 (4.5)	6 (35.3)	**0.02**
**Liver disease**				
HCC surveillance indication (yes, %)^2^	18 (50.0)	10 (47.6)	8 (44.4)	0.96
Treatment indication (yes, %)^2^	6 (16.7)	3 (13.6)	3 (16.7)	0.73
Significant liver fibrosis^4^	3 (13.6)	–	3 (16.7)	–
**HBV clinical phase**^3^				
ALT > ULN (%)^5^	8 (22.2)	4 (19.0)	4 (26.7)	0.59
HBV DNA > 2,000 lU/mL (%)	13 (36.1)	7 (33.3)	6 (42.9)	0.57
HBV DNA > 20,000 IU/mL (%)	7 (19.4)	3 (14.3)	4 (28.6)	0.3
HBeAg (%)	2 (5.1)	–	2 (11.8)	–

^1^ Linked to care = specialist care follow-up with HBsAg, ALT and HBV DNA test results and at least 1 abdominal ultrasound. ^2,3^ Data available for 90% and 92% of patierns, respectively. ^4^ detected using shear wave elastography, 6 kPa or above [25]. ^5^ Upper Limit of Normal ALT: 41 U/L for males, 31 U/L for females. ^6^ POCT: Point-of-care testing.

The costs associated with screening are shown in detail in Table 3.

**Table 3:** Overall costs of venepuncture and point-of-care testing (POCT) screening (in euros).

		Venepuncture	POC

**Personnel**	Nursing staff	3634.0	0.0
	Administrative assistant	1157.7	0.0
	Language services	160.0	0.0
	Study coordinator	1719.8	1013.5
	Physicians	4366.8	0.0
	TOTAL	11038.3	1013.5
**Logistics**	Blood tubes, venepuncture materials	4737.2	46.2
	Serological tests: HBsAg	7332.5	254.9
	Event logistics (location rent, catering, etc)	500.0	0.0
	Communication costs	1211.0	0.0
	TOTAL	13780.7	301.1
OVERALL COST		24819.0	1314.6
Screening uptake		100%	88.8%
**ADJUSTED COST**		**24819.0**	**1461.8**

**Figure 3:**
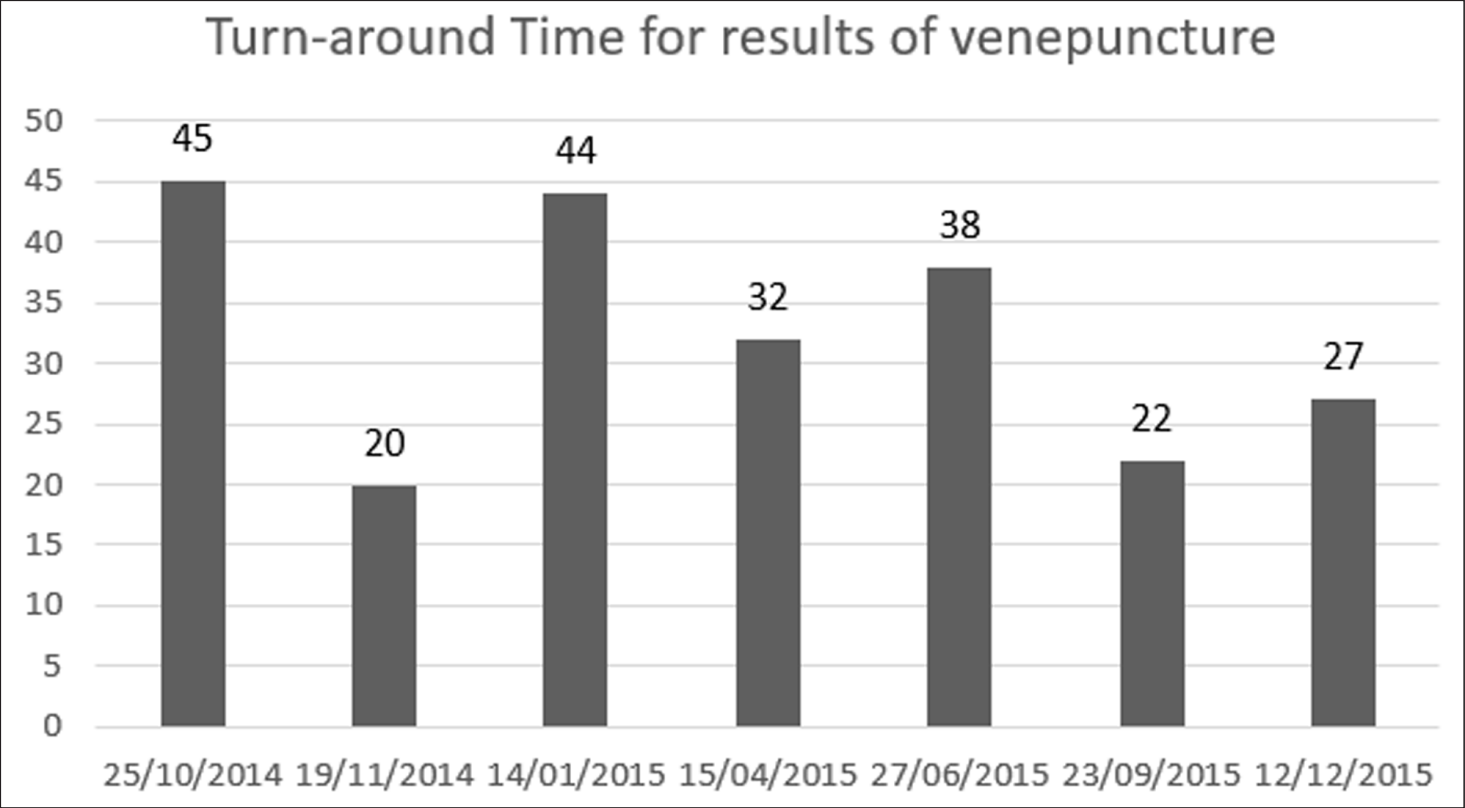
Cost comparison per patient screened (in euros).

Overall, the costs for screening were € 24,819 and € 2,750 for venepuncture and POC testing respectively. Taking into account the number of persons screened, the screening cost per person is € 54.0 and € 11.4 for venepuncture and POC testing respectively (Figure 3). Screening uptake differed between the two screening methods. While screening uptake was 100% when screening with venepuncture, uptake was 88.8% when using POC testing. Taking this into account, per person screening cost of the POC testing is € 12.7. Total adjusted cost saving is €41.3 or 76.5%.

## Discussion

This study provides empirical proof that POC screening tests for viral hepatitis B are associated with a higher linkage to care in an Asian migrant population, despite a lower overall cost and lower cost per person screened.

The WHO recently launched its ambition to eliminate viral hepatitis B and C infections as a public health problem by 2030 [3]. Although diagnostic tests with high-performance characteristics [14,15] and effective antiviral treatments are available, attaining the ambitious WHO goal will depend on identifying currently unknown carriers and linking them to specialist care. Linkage to care remains suboptimal, certainly in populations where viral hepatitis is highly endemic. In our data, a pressing sign of the importance of linkage to care is that 7.7% (n = 3/39) and 25.6% (n = 10/39) of the identified HBV patients who had treatment and HCC surveillance indications respectively in the venepuncture program [22], were lost to follow-up (Table 2).

Interestingly, a recent study by Sehr et al [19]. used Markov modelling to predict that POC testing may decrease long-term complications due to chronic HBV infection and improve linkage to care. Our data empirically support the wider implementation of POC testing due to its higher linkage to care of 86% of identified patients.

Only one-third of HBsAg positive individuals were linked to care using venepuncture-based testing, a result corroborated by numerous other studies [9,11,17,18]. Multiple barriers towards screening and linkage to care in regards to viral hepatitis exist in the Chinese community. A recent qualitative study by Lee et al [12]. highlights determinants in knowledge, cultural beliefs, social stigma, awareness, and views towards Western health systems as major contributors as to why viral hepatitis is an “invisible disease in an invisible population”. Barriers to care have also been investigated in other populations and HCV, HIV, and testing of sexually transmissible diseases [8,13]. Linkage to care was enhanced by facilitating referral for HCV assessment and scheduling of specialist appointments for screened persons. Barriers described in HIV testing included transport costs and distance, stigma, and fear of disclosure–factors similar to those described in screening for viral hepatitis in Asian populations.

Finally, we observed an overall HBsAg seroprevalence of 6.8% in the Asian, predominantly Chinese migrant community in three major cities in Belgium, which is in line with similar studies from the UK and the Netherlands [5,6,7].

Many potential issues and limitations must be addressed in our study.

First, it could be argued that the samples of both screening methods are not the same: venepuncture and POC tests were not randomised side by side at each screening site. However, the lack of statistical difference in seroprevalence of HBsAg between both protocols provides indirect evidence on the contrary. Also, when observing screened persons’ characteristics as a whole and comparing positively screened individuals based on their linkage to care (Table 2), all described characteristics showed no statistically significant difference across both groups except the screening method used (p = 0.020).

Second, while this study focused on the effect of POC testing on linkage to care, other factors besides the test modality differed between screening protocols. During venepuncture screening, persons were specifically motivated by community leaders and through peers to get tested. This may explain the observed higher screening uptake. POC testing was mostly an opportunistic screening during classes/professional activities. As opportunistic screening generally has a lower impact [23], the higher linkage to care during POC testing further underlines the importance of providing on-site results to motivate patients for clinical follow-up.

Finally, an a priori power analysis for linkage to care was not possible. Using observed data, however, the estimated power surpasses 80%. The high prevalence of HBV in this population and their well-known low rate of linkage to care allowed investigating the impact of POCT on linkage to care. Such an impact would be harder to assess in Caucasians living in a low seroprevalence area and with generally higher linkage to care [20].

## Conclusion

In an Asian population with a high HBV prevalence, we found that screening based on POC tests results in lower costs per person screened (76.5%), and a 2.5 times higher linkage to care.

## Data Accessibility Statement

The (anonymized) datasets used and/or analysed during the current study are available from the corresponding author on reasonable request.
